# Microwave-assisted catalytic conversion of glycerol to lactic acid using a synergistic trimetallic oxide (NiO–CuO–ZnO) system

**DOI:** 10.1039/d5ra09545j

**Published:** 2026-07-06

**Authors:** Jamna Prasad Gujar, Bharat Modhera, Yash Jaiswal

**Affiliations:** a Department of Chemical Engineering, Maulana Azad National Institute of Technology Bhopal Madhya Pradesh – 462003 India modherab@manit.ac.in; b Department of Chemical Engineering, Faculty of Technology, Dharmsinh Desai University Nadiad 387001 India

## Abstract

The catalytic conversion of glycerol into lactic acid offers a sustainable route for biomass valorization, yet achieving high selectivity under mild conditions remains challenging. Using a dehydrogenation-rearrangement route, we created a synergistic trimetallic oxide (NiO–CuO–ZnO) catalyst for microwave-assisted glycerol transformation. The optimized composition (NiO_1_CuO_2_ZnO_1_) achieved ∼98% glycerol conversion and ∼90% lactic acid selectivity in alkaline media. Physicochemical characterization revealed distinct catalyst roles: Cu sites drive dehydrogenation, Ni stabilizes reaction intermediates, and ZnO provides basic sites critical for glycerol activation. Quantitative XRD, FTIR and XPS confirmed phase purity, crystallite size, and relevant surface species. Using response surface methods and ANOVA validation, experimental data demonstrated significant agreement with model predictions. According to kinetic study, microwave irradiation decreases apparent activation energy due to localized interfacial heating and increased energy coupling with polar molecules. Reusability tests verified structural stability and preservation of active sites using XRD and FTIR, with very little metal leaching reported. Collectively, our findings demonstrate that rational design of redox-basic synergistic catalysts, combined with microwave-assisted processing, provides an efficient and sustainable pathway for glycerol valorization to lactic acid.

## Abstract 2

The catalytic conversion of glycerol into lactic acid offers a sustainable route for biomass valorization, yet achieving high selectivity under mild conditions remains challenging.

## Graphical abstract

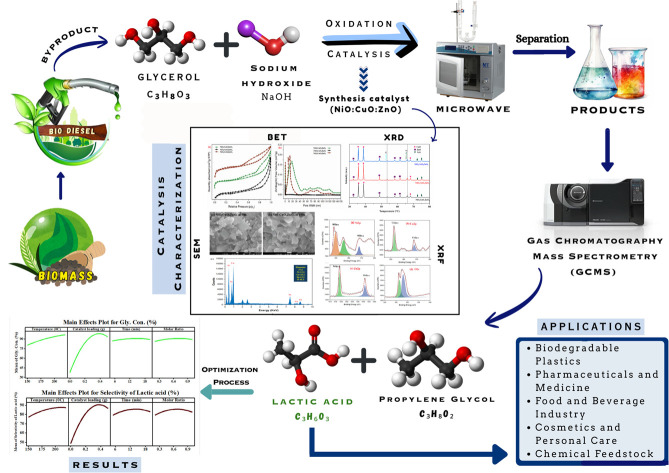

## 1. Introduction

The sustainable integration of natural resources in chemical processes remains a key challenge in addressing current environmental and energy issues. One example of this is biodiesel; this renewable fuel (which has other benefits, such as being biodegradable and having significantly lower greenhouse gas emissions compared to using fossil fuels) is produced from plants & animal fats. The process of making biodiesel creates a large quantity (nearly 10% of the total amount produced) of crude glycerol. This byproduct presents a challenge in waste management; however, it also provides an opportunity to create many more valuable chemicals through catalysts. The use of waste glycerol to produce other chemicals will be a good example of using the upcycling principles associated with the circular economy in biodiesel systems to develop sustainable carbon-neutral biodiesel systems over time.^1–6^ Oxidative transformation of glycerol to glyceric acid,^7^ its dehydration to acrolein,^8^ and hydrogenation to methanol have all been successfully reported, highlighting the versatility of this platform molecule. Currently, however, the refined glycerol feedstock has been the only source used for these processes, and it is costly and very much in demand for other uses. As a result, the dependence on a refined feedstock presents a significant limitation because the economic viability of these methods is impacted by both the high price of glycerol and its competing value.^9–11^ In this work we report a new reaction of glycerol for the synthesis of lactic acid (LA) and alkyl lactates that is applicable to waste reutilization and sustainable chemistry endeavors. Due to its use in food, medicine, and environmentally friendly plastics, LA is a crucial intermediary for biodegradable polylactic acid (PLA).^12^ Low-toxicity solvents that potentially take the place of petrochemicals are alkyl lactates.

**Scheme 1:**
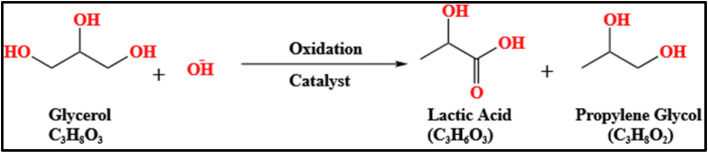
Lactic acid and PG production from glycerol.

Catalytic conversion proves to be a phenomenal asset for the framework of the circular economy. Biodiesel's viability has proliferated as a renewable energy source, also pushing the chemical industry closer to decarbonization by reducing excess glycerol and replacing fossil feedstocks.^13–16^ Researchers have accomplished significant progress in recent years, in catalytic glycerol valorization. Catalysts formed using metal-oxides, especially those precisely tuned, are highly effective in conversion of glycerol to lactic acid. Yang and colleagues, for example, achieved 94.6% selectivity for lactic acid under controlled circumstances by using a 30% CuO/ZrO_2_ catalyst.^17^ Zhang's team employed a different approach, achieving complete conversion and 83.2% lactic acid generation in less than an hour at 220 °C using a Cu–ZnO@C catalyst.^18^ These findings emphasise copper-based oxides' ability to selectively cleave and oxidize C–C bonds. Support materials play an important role. Torres and coworkers discovered that Co_3_O_4_/CeO_2_ outperformed other cobalt-based systems, citing increased oxygen mobility and acid-base synergy.^19^ On the engineering front, continuous-flow systems are driving forward. For example, Mimura's group used an Au–Pt/Al_2_O_3_ reactor to convert over 90% of glycerol and produce both glyceric and lactic acids on a large scale.^20^ However, issues remain. Traditional alkali-catalyzed techniques achieve 84.5% lactic acid yield with 2.5 M glycerol, but they have numerous limitations, including reactor corrosion and challenging energy-intensive separations.^21^ To address these difficulties, Chen and colleagues used a fed-batch technique to reduce NaOH corrosion in stainless steel, but residual alkalinity persisted.^22^ Similarly, when hydrogenolysis was utilized to create propylene glycol from glycerol, identical sustainability difficulties persisted (Scheme 1). When run under compressed H_2_, Ru or Ni-based metallic catalysts can achieve propylene glycol (PG) yields of more than 80%; however, their dependency on extra hydrogen lowers both process economics and overall carbon efficiency.

New technologies for *in situ* H_2_ production (*e.g.* aqueous-phase reforming, catalytic transfer hydrogenolysis) are attractive. But these methods have low PG selectivity (<60%) and other side reactions.^23,24^ This continuing conundrum underlines a key knowledge gap: there is a pressing need for integrated catalysis technologies that avoid H_2_ dependence while maintaining the desired selectivity in functional-group transformations to achieve high PG yields.

New catalyst design has moved towards bimetallic and non-noble metal catalysts. Abdullah *et al.* report a 41.4% yield of LA when using 0.43 NiO/CaO and claim that this is due to the stabilisation of reaction intermediates at the Ni–CaO junction.^25^ Cu-promoted Pt/active carbon catalysts successfully increase glycerol oxidation under mild conditions,^26^ metallic copper is more active than its oxides for dehydrogenation due to better electron transport kinetics.^27^ Yin *et al.* successfully produced 90% LA yields using Ni/graphite, showing that non-noble metals can be used.^28^

The objective of this study is to develop and optimize a green catalytic process for the sustainable transformation of waste-derived glycerol into lactic acid (LA) and propylene glycol (PG) with trimetallic NiO–CuO–ZnO mixed oxides. The catalyst is tailored to take advantage of synergy between metal and metal oxide that increases the redox activity as well as stability and selectivity for product. Structure–function relationships are established because of comprehensive physicochemical characterization (XRD, FTIR, BET, FE-SEM, TGA, XPS and Raman effect TPR/TPD). In the present work, reaction conditions including temperature, alkaline condition and catalyst dosage are carefully studied to achieve higher LA yield. The possibility of recycling catalyst is discussed with respect to the long-term operability of this technique. Uniqueness of the work including first time use catalyst NiO–CuO–ZnO this molar ratio and hydrogen-free route. In this context, a hydrogen-free and cost-effective strategy (objectives of circular bioeconomy) is proposed in this work.

## 2. Experiment section

### 2.1. Materials

Glycerol (purity > 99%) was procured from Merck, India. The metal precursors, including copper(ii) nitrate trihydrate (Cu (NO_3_)_2_ 3H_2_O), nickel(ii) nitrate hexahydrate (Ni (NO_3_)_2_ 6H_2_O), and zinc(ii) nitrate hexahydrate (Zn ((NO_3_)_2_ 6H_2_O)), were also obtained from Merck, India. Sodium carbonate (Na_2_CO_3_, 99.5%) was used as the precipitating agent. All chemicals were of analytical grade and used without further purification. Deionized water, obtained from the laboratory's water purification system, was used throughout the synthesis process.

### 2.2. Synthesis of NiO–CuO–ZnO composite metal oxides

**Fig. 1:**
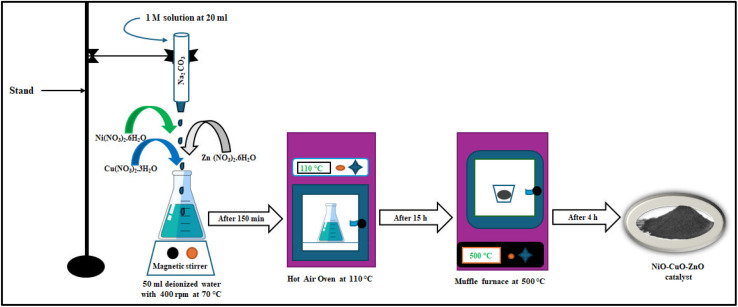
Synthesis of trimetallic oxide catalyst (NiO–CuO–ZnO) by wet chemical method.

Trimetallic NiO–CuO–ZnO composites with five distinct molar ratios (NiO–CuO–ZnO = 1 : 1 : 1, 2 : 1 : 1, 1 : 1 : 2, 1 : 2 : 1, and 2 : 1 : 2) were synthesized *via* a controlled co-precipitation route to optimize synergistic interactions for catalytic glycerol valorization. Stoichiometric aqueous solutions of Cu(NO_3_)_2_ 3H_2_O, Ni(NO_3_)_2_ 6H_2_O, and Zn(NO_3_)_2_ 6H_2_O were prepared double-deionized water and homogenized at 70 °C under continuous 500 rpm magnetic stirring. A few drops of CH_3_COOH were added to ensure a clean and consistent solution, resulting in the precipitation of mixed metal hydroxides. The resulting slurry was aged for 2 h at 30 °C to promote crystallite stabilization, followed by vacuum filtration and repeated washing with deionized water to remove residual ions. The precipitate was subsequently dried at 80 °C for 15 h and calcined at 500 °C for 4 h in static air to ensure complete conversion into oxide phases. The obtained catalysts were designated as NiO_1_CuO_1_ZnO_1_, NiO_2_CuO_1_ZnO_1_, NiO_1_CuO_1_ZnO_2_, NiO_1_CuO_2_ZnO_1_, and NiO_2_CuO_1_ZnO_2_ (Fig. 1).

### 2.3. Catalyst characterization

A wide range of analytical tools was utilized to explore the structural and physicochemical characteristics of the NiO–CuO–ZnO catalysts. The crystalline phases were examined by X-ray diffraction (XRD) using a RIGAKU-Japan (MINIFLEX) diffractometer with Cu-Kα radiation (*λ* = 1.54316 Å). Diffraction patterns were recorded in the 2*θ* range of 30–80°, with a step size of 0.02° and a scanning rate of 2° per min. Fourier-transform infrared (FTIR) spectroscopy was performed on a Shimadzu IR Affinity-1S instrument (Japan) at a resolution of 8 cm^−1^ with 64 scans to identify the chemical bonds and functional groups within the mixed oxide matrix. The Brunauer–Emmett–Teller (BET) surface area and pore characteristics were determined by nitrogen adsorption–desorption at liquid nitrogen temperature using a Quantachrome (USA) surface analyzer. Hydrogen temperature-programmed reduction (H_2_-TPR) and carbon dioxide temperature-programmed desorption (CO_2_-TPD) analyses were carried out on a Chem BET TP-5056 system (Quantachrome, USA) equipped with a thermal conductivity detector to study the redox behavior and surface basicity, respectively. Surface morphology and elemental distribution were analyzed using a TESCAN CLARA scanning electron microscope (SEM) equipped with energy-dispersive X-ray spectroscopy (EDX). The system operates at 0.2–30 keV, provides a magnification range of 20× to 20 000 00×, and achieves an SEI resolution of 0.9 nm at 15 keV. Thermal stability was evaluated through thermogravimetric analysis (TGA) on a Mettler Toledo TGA/DSC 1 HT 1600 instrument by heating approximately 5 mg of sample from ambient temperature to 1000 °C at 5 °C per min. The surface oxidation states of copper and other metal species before and after the catalytic reaction were investigated through X-ray photoelectron spectroscopy (XPS) using a Thermo Fisher ESCALAB 250 Xi analyzer, and the collected spectra were processed using Thermo Avantage software. Raman spectroscopy is an analytical method that uses scattered light to study the vibrational energy modes of materials. The CTR 300-S/C-x (TechnoS Instruments, Jaipur) confocal Raman microscope is equipped with 532 nm and 785 nm lasers, a Czerny–Turner spectrograph with three gratings, and a 10 µm–3.0 mm slit. It offers <0.1 cm^−1^ repeatability, 50–5000 cm^−1^ Raman shift range and includes fibre-optic and PC-based data software.

### 2.4. Catalytic transformation of glycerol to lactic acid *via* dehydrogenation rearrangement pathway

The catalytic transformation of glycerol to lactic acid was carried out in a microwave-assisted batch reactor under alkaline conditions, following a base-catalyzed dehydrogenation-rearrangement mechanism. The NiO–CuO–ZnO trimetallic catalyst provides synergistic redox–basic sites that facilitate glycerol activation, intermediate stabilization, and selective rearrangement toward lactic acid.

#### 2.4.1. Microwave reactor configuration and operating conditions

The reactions were carried out in an RG34L-series microwave reactor operating at 2.45 GHz and producing up to 490 W of power output. It had adjustable microwave power (100–720 W), stirring (1200 rpm), internal temperature detection with a fiber optic probe, and a closed vessel head design that enabled autogenous pressure to develop while avoiding evaporation.

It featured feedback coupling between the probe and the power which allowed precise control of the temperature and avoided hot spots. The use of typical stirring at 500 rpm promoted heat and mass transfer and avoided possible microwave-induced hot spots.

#### 2.4.2. Reaction procedure

We began with an aqueous solution containing 10% glycerol, 50 mg of our NiO–CuO–ZnO catalyst, and sodium hydroxide as a base (glycerol-to-NaOH molar ratio 1 : 1 to 1 : 5). Reactions were then carried out at temperatures between 150 and 220 °C for reaction times of 5 to 20 min. Under very alkaline circumstances (pH 12.5–13.8), glycerol is deprotonated to glycerolate, then Cu-mediated dehydrogenation to glyceraldehyde, followed by retro-aldol cleavage to pyruvaldehyde, and finally rearrangement to lactate, which yields lactic acid when protonated. The high pH neutralises side reactions that result in the dehydration products acrolein and acetol and thus promotes higher lactic acid yields.^29^

#### 2.4.3. Conventional heating comparison

Comparative experiments were conducted using conventional heating in a 100 mL three-neck round-bottom flask fitted with a reflux condenser, thermocouple and magnetic stirring (200–600 rpm). These experiments were carried out under the same reaction conditions to evaluate the influence of microwaves on the reaction.

#### 2.4.4. Catalyst separation, reuse, and product preparation

When the reaction was finished, it was allowed to cool to room temperature. The catalyst was filtered using vacuum filtration apparatus with Whatman (No. 42) filter paper, washed with ethanol–water (1 : 1, v : v) and dried at 80 °C and re-used in the subsequent catalytic tests. The liquid product was stabilized in the standard way and then diluted with a suitable amount of deionized water and methanol for analysis.^30^

#### 2.4.5. Gas chromatography–mass spectroscopy analysis and quantification

Analysis of the products was performed on a Shimadzu QP-2020 GC-MS system, equipped with an FID and capillary column SH-RXI-5SIL MS, (30-m length, ×0.25 mm ID × 0.25 µm film thickness). The GC oven temperature procedures include the following steps: the first oven temperature was 70 °C (held for 2 min). Then, a linear climb to 120 °C was carried out at a heating rate of 10 °C min^−1^, followed by a 4 min hold. The temperature was ramped up at 10 °C min^−1^ for 5 min until it reached 280 °C. Helium (99.99%) was employed as the carrier gas at a flow rate of 1.2 mL min^−1^. The analytical setup kept the ion source at 230 °C and the detector at 250 °C. The MS instrument was set to scan a mass range of 40 to 450 daltons (Da). Electron ionization (EI) was employed as the mode of ionization, and 0.5 µL (capacity of 10 µL) of each sample was injected, split ratio 2 : 1, ionization energy −70 eV, MS quadrupoles temperature 50 °C, SIM mode; and quantification peak area by SIM. The compounds were identified by comparing the mass spectra obtained with the NIST-11 and NIST-14 libraries, allowing for effective confirmation of the reaction's results.

#### 2.4.6. Quantitative analysis and calibration

The conversion of glycerol and selectivity to products was determined using the internal standard method, *n*-butanol. Standard solutions of glycerol and lactic acid were used to produce calibration curves and linear regression showed correlation coefficients of 0.99 or greater, indicating the analytical measurements were accurate. Using this method, glycerol conversion and lactic acid production were accurately established for all product evaluations.

#### 2.4.7. Detection limits and reproducibility

**Fig. 2:**
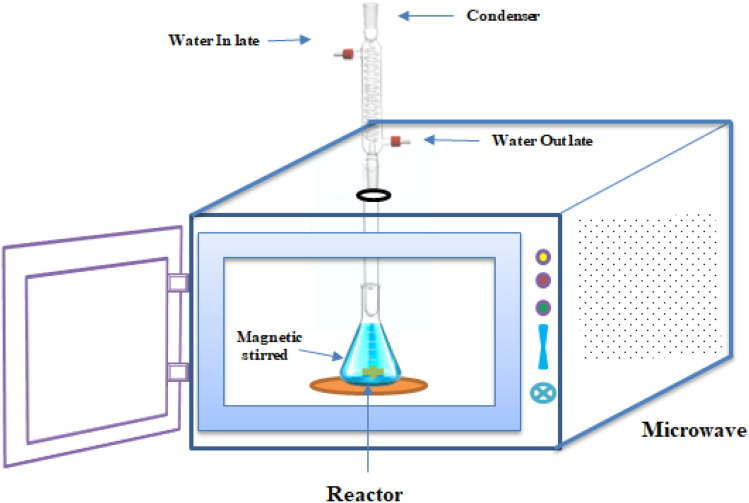
Synthesis of lactic acid from glycerol using microwave.

The limit of detection (LOD) and limit of quantification (LOQ) were around 0.5 wt% and 1.5 wt%, respectively. The experiments were conducted in triplicate, and the results were averaged. The uncertainty in the experimental data is in the range ±2%, suggesting good reproducibility in the whole set of data. Fig. 2 shows the experimental set-up.

#### 2.4.8. Performance evaluation

The glycerol conversion (*X*) and product selectivity (*S*) obtained from the reaction were computed respectively according to eqn (1) and (2):1
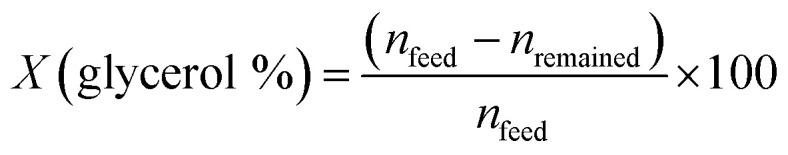
2
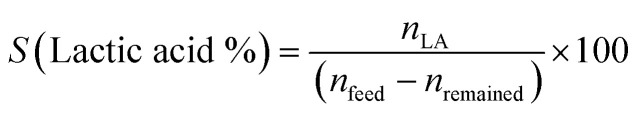
where *n*_feed_ and *n*_remained_ are the initial and remaining molar quantity of glycerol, respectively, *n*_LA_ is the molar quantity of produced lactic acid.

## 3. Results and discussion

### 3.1. Characterization of catalyst NiO–CuO–ZnO composite

#### 3.1.1. X-ray diffraction analysis

**Fig. 3:**
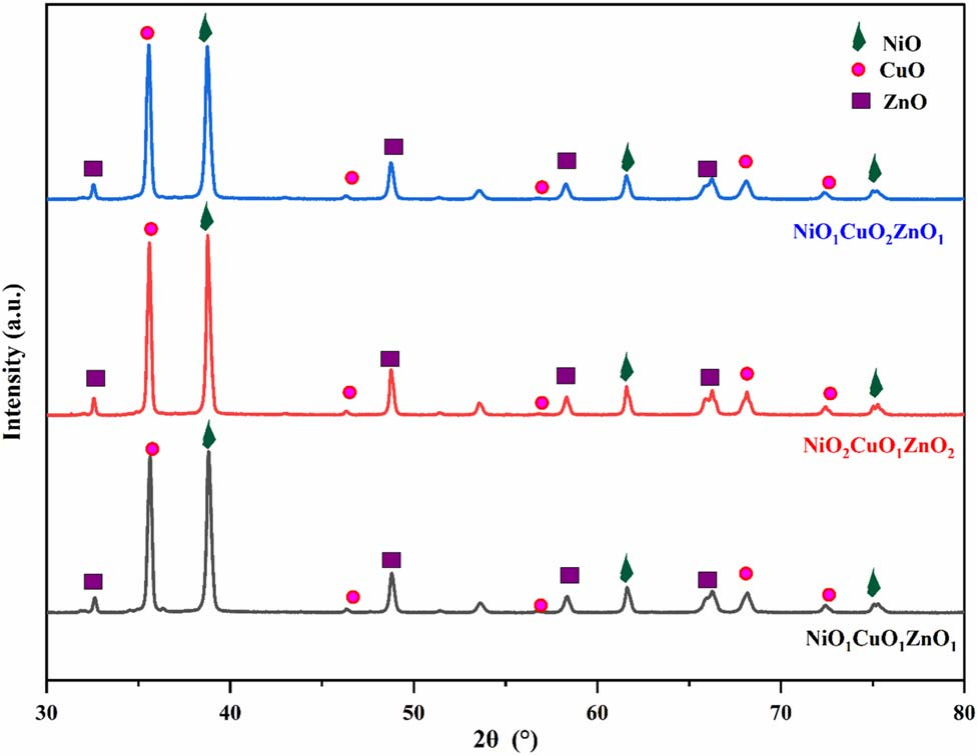
XRD analysis of NiO–CuO–ZnO catalyst was pre-reaction.

X-ray diffraction (XRD) analysis was employed to investigate the crystalline structure and phase composition of the NiO–CuO–ZnO trimetallic catalyst. The diffraction patterns confirm the coexistence of three distinct oxide phases without the formation of any impurity phases, indicating successful synthesis of a mixed-oxide system.^31^ The distinctive diffraction peaks belonging to cubic NiO (JCPDS 71-1179) were detected at 2*θ* = 38.7°, 61.8°, and 75.4°, while peaks at 36.5°, 46.3°, 56.7°, 68.8°, and 72.3° were assigned to monoclinic CuO (Fig. 3). Reflections of 31.8°, 48.6°, 58.27°, and 66.24° indicated the presence of hexagonal wurtzite ZnO (JCPDS 79-0205). No additional peaks were observed which confirms phase purity and no parasitic phases were formed during the synthesis process.^32,33^ The Scherrer equation was used to calculate the crystallite size and was evaluated for the most prominent peaks:3
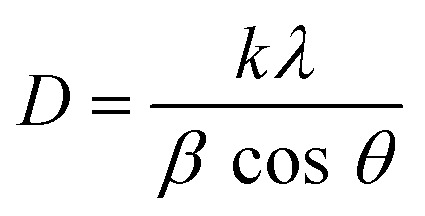
where *D* is the crystallite size, *K* is the shape factor (0.9), *λ* is the X-ray wavelength (1.5406 Å), *β* is the full width at half maximum (FWHM) and *θ* is the Bragg angle.

The average crystallite sizes obtained were around 12–25 nm, which confirm that the catalyst is nanocrystalline. The small crystallite size and broadened peaks indicate a high degree of dispersion of the active phases and intimate contact between NiO, CuO and ZnO phases. These characteristics are essential for catalytic activities, as they increase active sites and improve electron transfer across interfaces. From a catalytic perspective, the coexistence of these phases provides a synergistic framework, where:

• CuO acts as a redox-active site facilitating glycerol dehydrogenation,

• NiO contributes to intermediate stabilization, and,

• ZnO enhances surface basicity and promotes rearrangement reactions.

Thus, the XRD results not only confirm phase formation but also support the structure–activity relationship, linking nanoscale crystallinity and phase synergy to improved catalytic performance in glycerol-to-lactic acid conversion.

#### 3.1.2. FTIR analysis

**Fig. 4:**
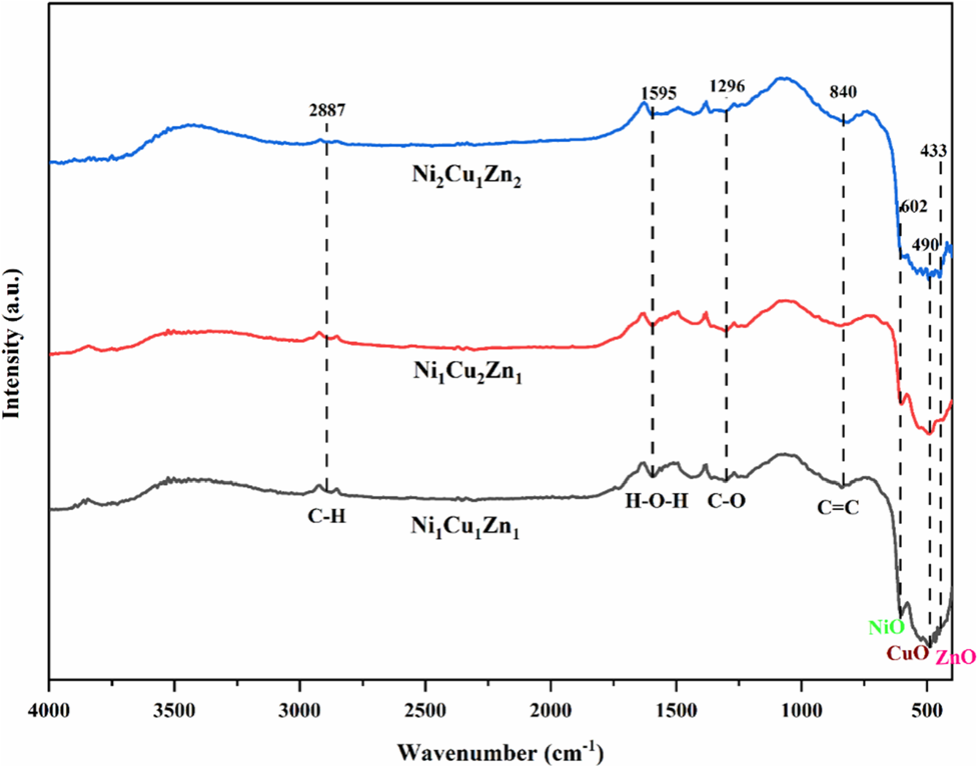
FTIR analysis of NiO–CuO–ZnO catalyst was pre-reaction.

FTIR spectroscopy method confirmed that the main functional groups exist in the NiO–CuO–ZnO catalyst samples. FTIR analysis took place before any reaction occurred. FTIR spectra in Fig. 4 show multiple distinct absorption bands. The catalyst surface contains hydroxyl groups which produce a wide peak that spans from 3250 to 3600 cm^−1^. The C–H stretching vibrations from organic residues produce a distinct band which appears at 2887 cm^−1^ in all samples. The H–O–H bending vibrations between 1595–1630 cm^−1^ indicate that water and hydroxyl groups exist as surface adsorbates. The surface functionalities play a crucial role because they enable glycerol activation and adsorption during catalytic reactions. FTIR peaks at 1296 cm^−1^ and 840 cm^−1^ indicate the presence of organic compounds including lactic acid derivatives which form during the catalytic process according to post-reaction samples. In the lower wavenumber region (450–1000 cm^−1^), narrow peaks were attributed to metal–oxygen (M–O) bonds. Within the fingerprint region (400–700 cm^−1^), distinct absorptions at 602 cm^−1^, 490 ^−1^, and 433 cm^−1^ confirmed the incorporation of Ni–O, Cu–O, and Zn–O bonds, respectively. Furthermore, peaks in the ranges of 800–930 cm^−1^, 600–750 cm^−1^ and 450–570 cm^−1^ were also assigned to Zn–O, Ni–O, and Cu–O vibrations, consistent with previous reports.^34,35^

The intensity of the system's absorption bands was directly proportional to the molar ratio of the relevant metals in the CuO–NiO–ZnO system. The results validate the catalysts' structural stability and surface functional groups while also confirming the XPS results from section 3.1.7, which are required for excellent catalytic performance and selective lactic acid production during glycerol oxidation.

#### 3.1.3. Textural and surface properties

**Fig. 5:**
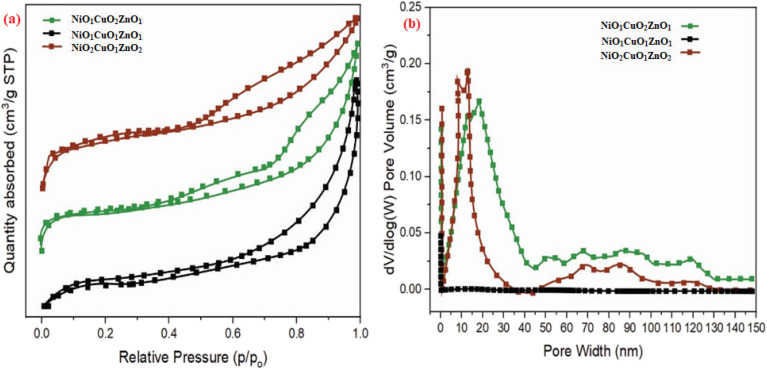
(a) BET analysis and (b) pore size distribution of NiO–CuO–ZnO catalyst.

The nitrogen adsorption–desorption isotherms of all NiO–CuO–ZnO samples showed Type IV characteristics with H3-type hysteresis loops which indicated their mesoporous structures (Fig. 5). The sample NiO_1_CuO_2_ZnO_1_ achieved the highest BET surface area of 124 m^2^ g^−1^ in Fig. 5(a) and (b) maintained an optimal pore structure (0.21 cm^3^ g^−1^, 18.2 nm) which enabled better active site distribution and better mass transport for glycerol adsorption and dehydrogenation reactions. The combination of these characteristics in NiO_1_CuO_2_ZnO_1_ makes it the best catalyst for lactic acid conversion because it shows superior performance compared to other compositions under optimal conditions (Table 1). The BJH pore-size distribution of the NiO_2_CuO_1_ZnO_2_ catalyst shows a peak at 15 nm which indicates its narrow and uniform mesoporous structure that enhances catalytic mass transport.

**Table 1:** BET analysis of NiO–CuO–ZnO different ratio

Catalyst composition	BET surface area (m^2^ g^−1^)	Pore volume (cm^3^ g^−1^)	Average pore diameter (nm)
NiO_1_CuO_1_ZnO_1_	112	0.18	16.5
NiO_1_CuO_2_ZnO_1_	124	0.21	18.2
NiO_2_CuO_1_ZnO_2_	120	0.25	20.3

#### 3.1.4. H_2_-TPR and CO_2_-TPD profiles

**Fig. 6:**
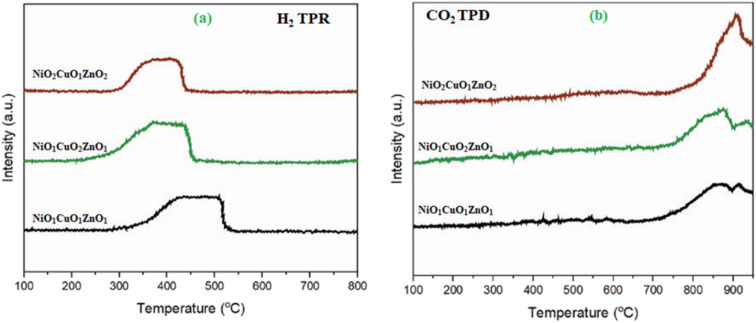
(a) H_2_ TPR and (b) CO_2_ TPD analysis of NiO–CuO–ZnO catalyst.

The results from H_2_-TPR and CO_2_-TPD show how the addition of Cu and Zn changes the redox and basic properties of NiO–CuO–ZnO catalyst series. In Fig. 6(a), the NiO_2_CuO_1_ZnO_2_ sample exhibits two distinct reduction peaks, one at around 360 °C and another at around 500 °C. These peaks correspond to the reduction of highly dispersed CuO and highly interacting NiO–ZnO species, respectively. This suggests improved metal-support interactions and enhanced reducibility compared to the equimolar NiO_1_CuO_1_ZnO_1_ catalyst, which exhibits a wide peak around 430 °C, signalling the reduction of bulk CuO. These results are consistent with Zhang *et al.* (2023), which showed that Zn addition enhances the dispersion of Cu- and Ni-based oxides, and their reduction temperature becomes lower.^18^

In Fig. 6(b), the CO_2_-TPD measurements show differences in the surface basicity of the catalysts. The NiO_2_CuO_1_ZnO_2_ sample, with its high zinc content, has a distinct peak around 220 °C and a broad band at higher temperatures (350 °C). These bands indicate that medium-to-strong basic sites are much more abundant. These sites are crucial for deprotonating glycerol, stabilizing glycerolate intermediates, and promoting the rearrangements that lead to lactic acid formation. Our studies are centred on the role of basicity, but we recognize that many transition-metal oxides also contain Lewis's acid sites. A qualitative NH_3_-TPD assessment (or reference to literature acidity trends) suggests that the relative acidity of the prepared samples is low compared with their basic character, and the presence of ZnO further diminishes strong Lewis's acidity. This aligns with the observed suppression of dehydration by-products and the enhanced selectivity toward lactic acid under alkaline reaction conditions.

#### 3.1.5. FE-SEM analysis

**Fig. 7:**
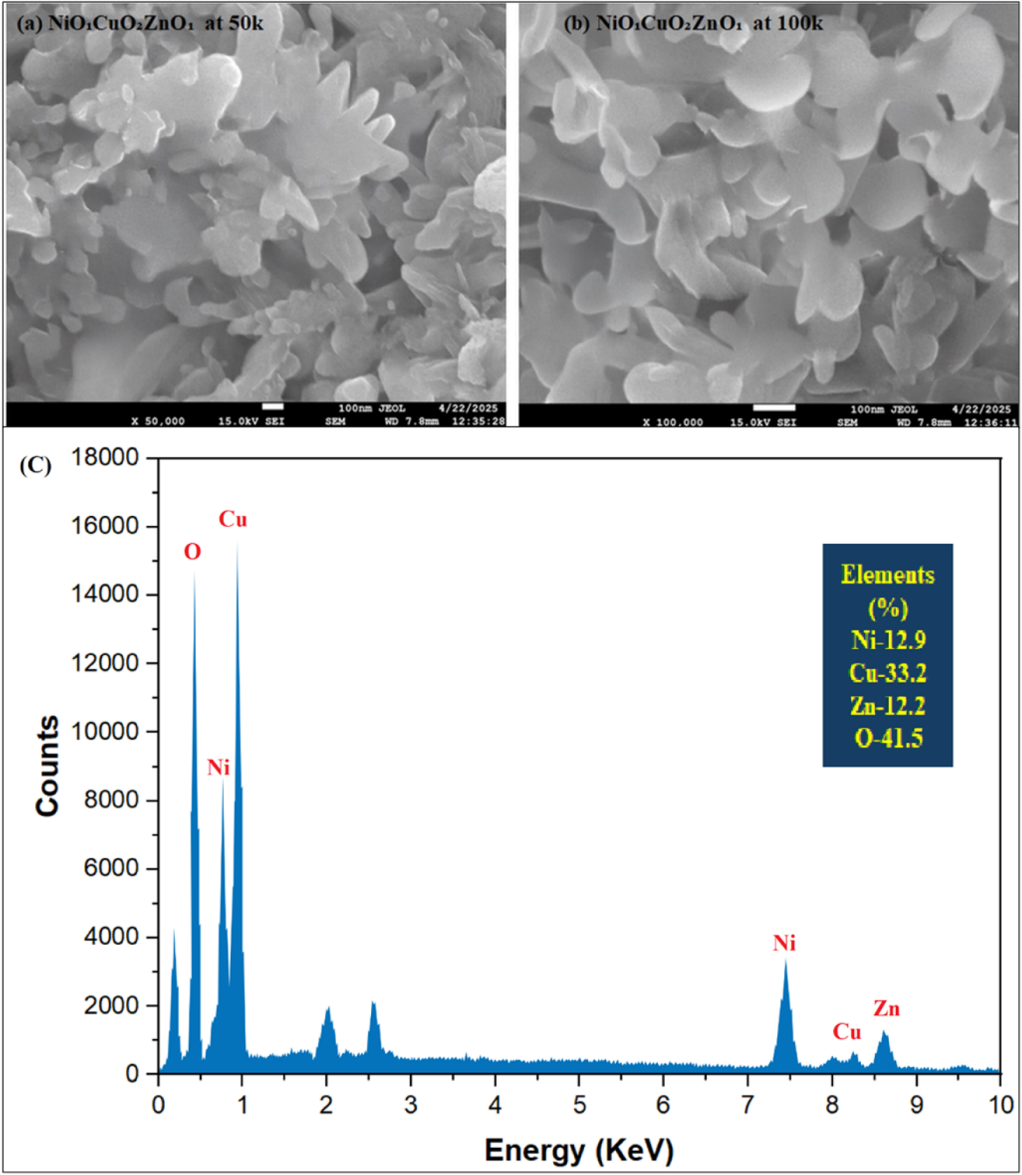
FE-SEM image (a) NiO_1_: CuO_2_: ZnO_1_ at 50k and (b) NiO_1_: CuO_2_: ZnO_1_ at 100k and (c) EDX analysis of NiO_1_: CuO_2_: ZnO_1_ catalyst after analysis 500 °C.

FE-SEM analysis was used to explore the morphology and microstructure of the CuO–NiO–ZnO catalyst synthesized in the present study. The representative images shown in Fig. 7(a and b) reveal that the nanoparticles are well dispersed and their boundary is well defined. It has been reported that particle shape development and its aggregation tendency depend strongly on precursor concentration, reaction time, solvent environment, and calcination protocol. In this work, calcination was performed at 500 °C. Energy-dispersive X-ray spectroscopy (EDX) elemental mapping revealed that Ni, Cu, and Zn distribution within the catalyst surface was highly uniform, pointing toward an effective dispersion of all three metal components. Quantitative EDX analysis (atomic %) yielded Ni = 12.9%, Cu = 33.2%, Zn = 12.2%, and O = 41.5% (Fig. 7(c)). The absence of extraneous peaks above the detection threshold further substantiates the compositional purity of the synthesized material.

EDX found a uniform elemental distribution while XRD showed phase selective diffraction peaks. These observations confirm the presence of CuO, NiO and ZnO as discrete, but intimately mixed phases, rather than large separate domains.^36^ The FE-SEM images and EDX spectra also support the XRD results regarding crystallite size and phase identification, suggesting a structurally and compositionally homogeneous trimetallic oxide with good morphology for catalysis. These include: a large surface area; accessible active sites for reactant interaction; and a good interfacial contact between the oxide phases, which is important for the reaction.

#### 3.1.6. Raman effect in NiO–CuO–ZnO composite catalyst

**Fig. 8:**
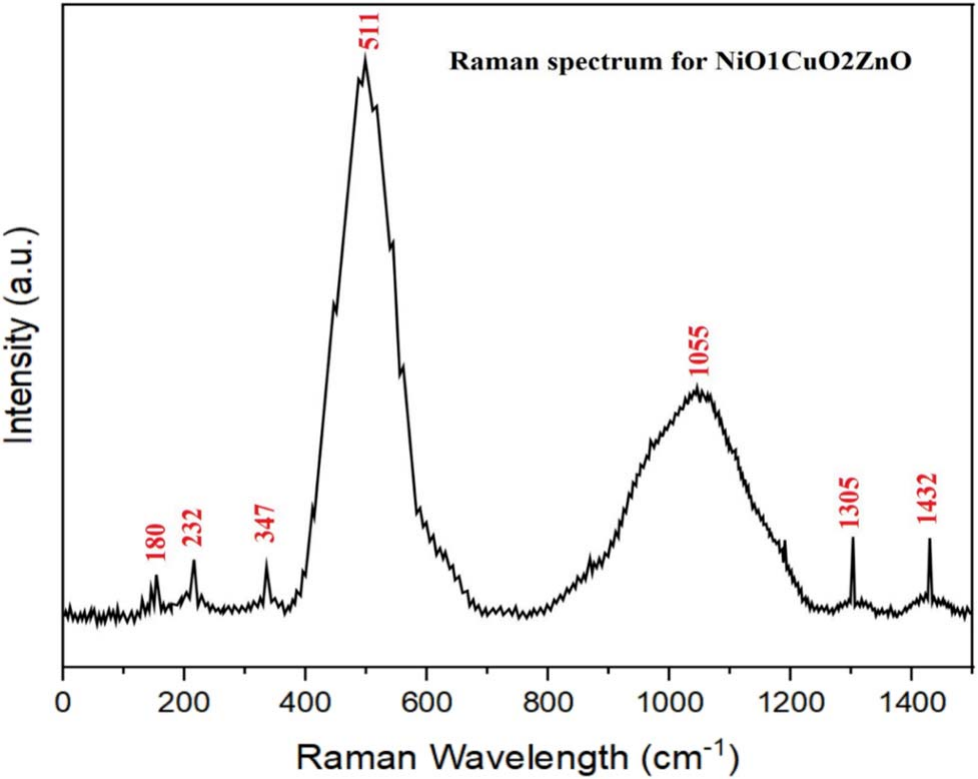
Raman spectrum of the NiO–CuO–ZnO composite after calcination at 500 °C.

Raman spectrum of NiO–CuO–ZnO mixed metal oxide composite annealed at 500 °C for 4 h shows multiple vibrational modes which prove the formation of a crystalline multi-phase catalyst structure. The 511 cm^−1^ peak shows strong lattice vibrations from ZnO or CuO while the 180 cm^−1^, 232 cm^−1^ and 347 cm^−1^ peaks represent NiO and CuO phonon modes which confirm metal oxide integration. The defect states and oxygen vacancies and multiphonon scattering events produce peaks that appear at 1055 cm^−1^, 1305 cm^−1^ and 1432 cm^−1^ which enhance catalytic performance. The peak-fitting method allowed researchers to separate multiple Raman signals which revealed the complex structure of the composite material and its possible active sites for catalytic reactions (Fig. 8). The XRD analysis shows three oxide phases which are confirmed by the overlapping Raman peaks.

#### 3.1.7. X-ray photoelectron spectroscopy (XPS) analysis

**Fig. 9:**
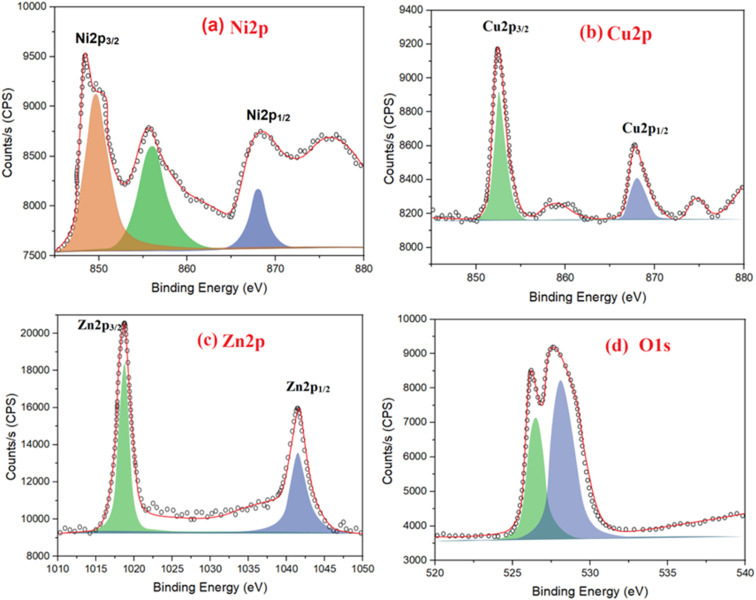
XPS analysis of catalyst NiO–CuO–ZnO. (a) Ni 2p, (b) Cu 2p, (c) Zn 2p and (d) O 1s region.

X-ray photoelectron spectroscopy (XPS) was employed to investigate the surface chemical composition, oxidation states, and electronic interactions within the NiO–CuO–ZnO catalyst (Fig. 9). The survey spectrum (0–1200 eV) confirms the presence of Ni, Cu, Zn, O, and C elements, consistent with the formation of a trimetallic oxide system.^37^

##### 3.1.7.1 Ni 2p region (Ni^2+^/Ni^3+^ states)

The Ni 2p high-resolution spectrum reveals the presence of a prominent Ni 2p_3/2_ peak at ∼855 eV and the Ni 2p_1/2_ peak at ∼872 eV, as well as shake-up satellites. These features are characteristic of Ni^2+^ species (NiO) with a small fraction of surface hydroxylated Ni (NiOH) species. The peak fit of Ni 2p signals indicates the presence of a majority of Ni^2+^ species (>85%), with small contribution from higher oxidation states or surface defects. The Ni^2+^ sites play a critical role in stabilizing key intermediates in solvent-free glycerol conversion, to avoid side reactions and optimize a selective transformation to lactic acid.

##### 3.1.7.2 Cu 2p region (Cu^+^/Cu^2+^ ratio)

The Cu 2p_3/2_ peak ranged from 932.5–934.5 eV, indicating Cu^+^/Cu^2+^ species. The presence of high intensity shake-up satellite peaks in the region 940–945 eV confirms dominance of Cu^2+^ (CuO) in the catalyst. Deconvolution analysis shows a Cu^2+/^Cu^+^ ratio of about 3 : 1, suggesting that Cu is mostly in an oxidized state and the remaining a small number of reduced species. The signal observed at a binding energy of ∼852 eV in the previous report is probably a result of inadequate experimental calibration or background anomaly, as it is not consistent with binding energy of Cu oxides. The new calibration result shows that Cu^2+^ is the dominant species. In terms of catalysis, Cu^2+^ is the active site for the dehydrogenation of glycerol and the presence of small quantities of Cu^+^ can affect the selectivity by modifying adsorption and electron transfer reactions of intermediates.

##### 3.1.7.3 Zn 2p region (Zn^2+^ in ZnO)

The existence of Zn^2+^ in ZnO is indicated by the Zn 2p_3/2_ and Zn 2p_1/2_ peaks around ∼1022 eV and ∼1045 eV, respectively. No satellite characteristics were seen. ZnO mainly provides Lewis basic sites, which facilitate glycerol deprotonation and isomerisation. In addition, ZnO is significant for Cu stabilisation and promoting interactions between metal oxides.

##### 3.1.7.4 O 1s region (lattice oxygen *vs.* hydroxyl species)

The O 1s spectrum exhibits two main components:

• ∼529.8–530.2 eV corresponding to lattice oxygen (O^2−^)

• ∼531–532 eV attributed to surface hydroxyl groups and adsorbed species.

A relatively high fraction of surface hydroxyl groups indicates the presence of defect-rich and hydroxylated surfaces, which are favorable for base-catalyzed reactions. These –OH groups are particularly important for facilitating glycerolate formation and subsequent rearrangement steps.

##### 3.1.7.5 Structure–activity relationship (XPS insight)

The XPS results reveal a synergistic interaction between metal oxides, where:

• Cu^2+^ sites enable dehydrogenation of glycerol,

• Ni^2+^ sites stabilize reaction intermediates and suppress side reactions,

• ZnO-derived basic sites promote rearrangement to lactate,

• Surface hydroxyl groups and oxygen vacancies enhance catalytic activity by facilitating adsorption and activation of reactants.

This combination of redox and basic functionalities explains the high glycerol conversion and lactic acid selectivity observed for the trimetallic catalyst.

#### 3.1.8. TGA analysis

**Fig. 10:**
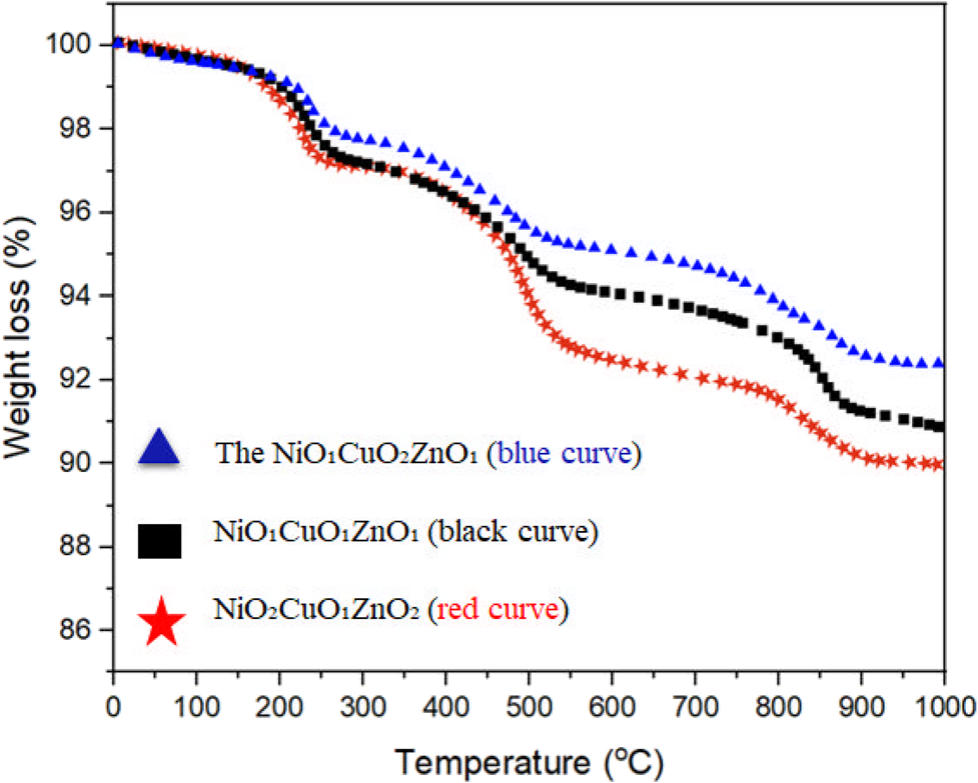
TGA analysis of NiO–CuO–ZnO catalyst and find the mass losses.

TGA assesses thermal stability and compositional changes in NiO–CuO–ZnO catalyst systems over a wide temperature range (0–1000 °C). As demonstrated in Fig. 10, all samples had two major weight decrease regions. Examination of the TGA profiles revealed two unique mass-loss regimes for all investigated compounds. The first mass loss at 100 °C (0.4–0.8%) is due to the desorption of residual physiosorbed water and low-boiling volatiles, indicating that the samples were pre-dried before analysis.^38^ Between ambient and 500 °C, the most significant mass decrement (4.7–7.0%) occurs due to stepwise de-hydroxylation, disintegration of organics or carbonate residues, and the commencement of metal oxide condensation processes.

Notably, the NiO_2_CuO_1_ZnO_2_ (red-labeled) specimen exhibits the greatest weight loss in this interval, implying a relatively high proportion of labile surface species or increased accessible surface, favoring volatile release (Table 2). The weight loss across all systems is recorded with a gradual and compositional dependence over the 500 °C–900 °C temperature interval at cumulative loss values of 7.8% for blue, 9% for black, and 10% for red. Extensive de-hydroxylation, possible oxidation, and/or slow reconfiguration/compaction of the oxide lattice due to high temperature phase transitions and densification account for the weight loss seen in this range. When the data are combined, they portray the NiO_1_CuO_2_ZnO_1_ formulation (blue) exhibiting better thermal stability compared to that of the NiO_2_CuO_1_ZnO_2_ formulation (red). Based upon the calcination temperature (500 °C) employed, it can be concluded that the calcination of these materials will lead to removal of most of the volatiles and weakly attached materials and that any differences in mass loss are attributed to the compositional and morphological properties of these materials on their thermal stability. The results show the need to optimize the composition when designing mixed metal oxide catalysts for their thermal stability under process conditions.

**Table 2:** TGA analysis of mass losses with different temperature

Sample (curve)	Remaining @100 °C (%)	Mass loss @100 °C (%)	Remaining @500 °C (%)	Mass loss @500 °C (%)	Remaining @900 °C (%)	Mass loss @900 °C (%)
NiO_1_CuO_2_ZnO_1_ (blue)	99.6	0.4	95.3	4.7	92.3	7.7
NiO_1_CuO_1_ZnO_1_ (black)	99.4	0.6	94.2	5.8	91.0	9.0
NiO_2_CuO_1_ZnO_2_ (red)	99.2	0.8	93.0	7.0	90.0	10.0

### 3.2. Effect of NiO–CuO–ZnO mole ratios

**Fig. 11:**
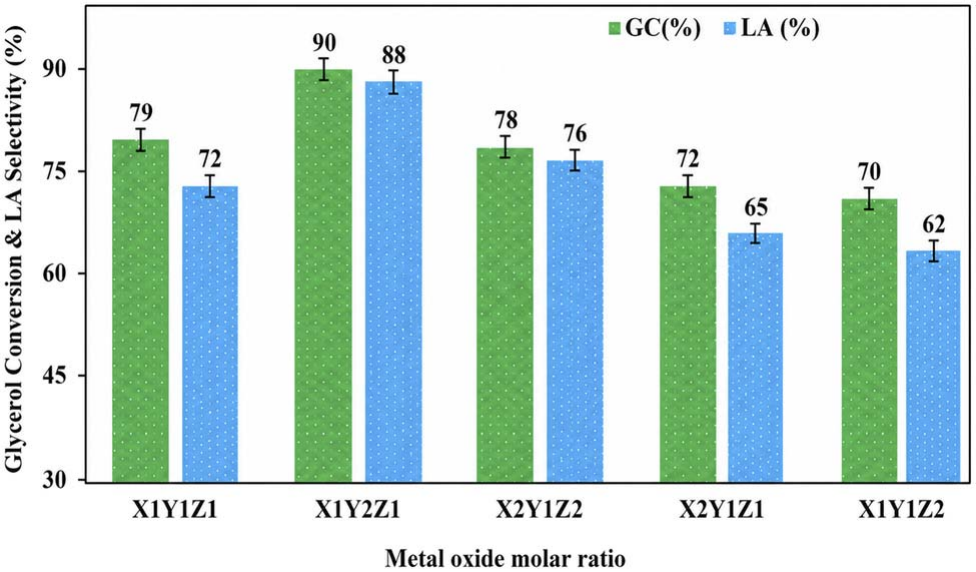
Effect of molar ratio of gly. con. and selectivity of LA (%). Reaction conditions: = 200 °C, 20 min, NaOH/glycerol = 1.1 mol mol^−1^, catalyst/glycerol = 0.027 g g^−1^.

The influence of NiO(x), CuO (*Y*), and ZnO (*Z*) molar ratios on glycerol conversion and lactic acid (LA) selectivity was systematically investigated, as presented in Fig. 11. Among the studied catalysts, NiO_1_CuO_2_ZnO_1_ exhibited the highest catalytic performance, achieving 90 ± 2% glycerol conversion with 88 ± 2% LA selectivity. The enhanced activity can be attributed to the optimized composition of metal oxides, which governs the surface acidity/basicity, dispersion of active species, and synergistic interactions among the catalytic components.

#### 3.2.1. Effect of composition on acidity/basicity

CO_2_-temperature programmed desorption (CO_2_-TPD) analysis revealed that ZnO contributes moderate to strong basic sites that facilitate glycerol deprotonation, enolate formation, and retro-aldol condensation during lactic acid formation. However, excessive ZnO loading results in overly strong basicity, promoting undesired side reactions and reducing LA selectivity, as observed for the NiO_2_CuO_1_ZnO_2_ catalyst. In contrast, insufficient ZnO content limits glycerol activation and suppresses intermediate formation. Therefore, an appropriate ZnO concentration is essential to balance catalytic activity and product selectivity.

#### 3.2.2. Effect of composition on metal dispersion and active site formation

The CuO content plays a critical role in governing the dispersion of active metal species and the formation of catalytically active interfacial sites. In the optimized NiO_1_CuO_2_ZnO_1_ catalyst, the higher CuO proportion promotes uniform dispersion of Cu species over the catalyst surface and facilitates the formation of well-dispersed Cu–Ni and Cu–Zn interfaces. These interfacial sites improve the accessibility of active centers and enhance electron transfer between the metal oxides, thereby accelerating glycerol dehydrogenation and subsequent conversion to lactic acid. In contrast, catalysts with lower CuO content (*e.g.*, NiO_1_CuO_1_ZnO_1_) possess fewer redox-active sites, resulting in lower catalytic efficiency. Similarly, excessive ZnO or NiO loading can partially dilute or cover the Cu active phase, decreasing Cu dispersion and weakening synergistic interactions among the metal oxides.

The catalytic functions of the individual oxides are also highly complementary. CuO primarily facilitates the initial dehydrogenation of glycerol to glyceraldehyde or dihydroxyacetone intermediates, while NiO contributes to intermediate stabilization and suppresses undesirable side reactions. Simultaneously, ZnO provides the basic sites necessary for glycerol activation, C–C bond cleavage, and rearrangement reactions leading to lactate formation. In the NiO_1_CuO_2_ZnO_1_ catalyst, these functionalities are optimally balanced, enabling efficient conversion of glycerol to lactic acid with minimal byproduct formation.

#### 3.2.3. Structure activity relationship

**Scheme 2:**
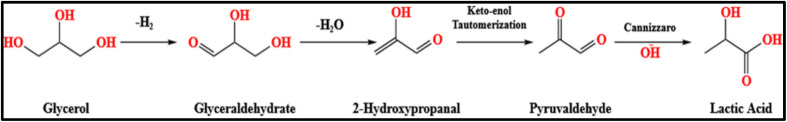
Reaction pathway of glycerol to lactic acid.

The superior catalytic performance of NiO_1_CuO_2_ZnO_1_ can, therefore, be explained through a strong structure–activity relationship. The optimized balance between redox-active CuO/NiO species and the basic nature of ZnO creates favorable catalytic interfaces that enhance electron exchange, stabilize reaction intermediates, and promote selective LA formation. In contrast, non-optimized compositions such as 1 : 1 : 1 or 2 : 1 : 2 exhibit either insufficient basicity, lower Cu active site density, or weaker interfacial synergy, resulting in reduced glycerol conversion and lower LA selectivity (Scheme 2).

#### 3.2.4. Implication of oxide tailoring

Overall, tuning the molar ratio of mixed metal oxides effectively controls the acid–base properties, active site density, and reaction pathway selectivity of the catalyst. These findings demonstrate that rational oxide composition engineering is a promising strategy not only for glycerol valorization but also for broader biomass-derived platform molecule upgrading applications.

### 3.3. Optimization of process parameters using response surface methodology

Response surface methodology (RSM) was used to systematically examine and optimize the critical process factors that influence glycerol conversion and selectivity of LA. A four-variable factor, one to three level experimental design was used, which included reaction temperature (*x*), reaction time (*y*), catalyst loading (*z*), and glycerol-to-NaOH molar ratio (*m*). A total number of 27 experimental runs were done using a full factorial design, and the results are shown in Table 3. It is vital to note that all data utilized in RSM modelling were gathered from experimentally performed reactions, with no data created or extrapolated from literary sources. To ensure reproducibility and statistical reliability, each experiment was repeated three times, and average values were utilized to create the model.

**Table 3:** Design of experiments dataset of glycerol conversion (%) to lactic acid (%)

Experiments	Temperature (°C)	Catalyst (g)	Time (min)	Molar ratio (gly: NaOH)	Glycerol conversion (%)	Selectivity lactic acid (%)
1	220	0.25	20	0.6	96.5	86.9
2	185	0.25	5.0	1.0	88.1	76.2
3	185	0.5	12.5	1.0	95.1	82.2
4	220	0.0	12.5	0.6	48.1	45.3
5	150	0.0	12.5	0.6	31.4	43.1
6	185	0.0	5.0	0.6	28.8	42.5
7	185	0.25	20	0.2	78.4	75.1
8	185	0.5	12.5	0.2	88.5	78.4
9	185	0.0	12.5	1.0	33.5	45.2
10	185	0.0	20	0.6	34.2	46.8
11	220	0.25	12.5	0.2	93.2	79.4
12	185	0.25	12.5	0.6	90.8	85.5
13	185	0.25	12.5	0.6	90.9	85.7
14	185	0.0	12.5	0.2	33.1	43.8
15	150	0.5	12.5	0.6	78.1	74.3
**16**	**220**	**0.5**	**12.5**	**0.6**	**98.4**	**89.8**
17	185	0.5	5.0	0.6	90.2	84.9
18	220	0.25	12.5	1.0	93.6	86.1
19	150	0.25	12.5	0.2	78.1	75.8
20	150	0.25	20	0.6	81.2	74.3
21	150	0.25	12.5	1.0	74.6	75.2
22	220	0.25	5.0	0.6	89.1	82.2
23	185	0.25	5.0	0.2	87.1	71.4
24	150	0.25	5.0	0.6	77.5	65.5
25	185	0.25	12.5	0.6	90.7	85.6
26	185	0.5	20	0.6	94.9	83.9
27	185	0.25	20	1.0	91.4	78.5

#### 3.3.1. Model development and regression analysis

The experimental data were fitted to a second-order polynomial equation to establish the relationship between independent variables and response functions (glycerol conversion and LA selectivity). The resulting regression models are expressed as:

(a) Glycerol conversion (%)*X* = −147.9 + 1.535 *x* + 385.3 *z* + 1.47 + 19.0 *m* − 0.003582 *x*^2^ − 517.2 *z*^2^ − 0.0456 *y*^2^ − 11.88 *m*^2^ + 0.103 *x* × *z* − 0.00219 *x* × *y* − 0.0375 *x* × *m* − 0.227 *z* × *y* + 3.0 *z* × *m* + 0.500 *y* × *m*.

(b) Selectivity of lactic acid (%)*S* = −12.5 + 0.523 *x* + 210.7 *z* + 0.129 *y*+ 6.6 *m* − 0.001378 *x*^2^ − 302.4 *z*^2^ − 0.0071 *y*^2^ − 17.27 *m*^2^ + 0.0657 *y* × *z* − 0.00010 *x* × *y* + 0.0768 *x* × *m* − 0.440 *z* × *y* + 23.50 *z* × *m* + 0.383 *y* × *m*.Note: *x* = temperature (°C), *y* = time (min), *z* = catalyst (g), *m* = G: NaOH molar ratio (m).

#### 3.3.2. Statistical validation of the model (ANOVA)

We used analysis of variance to check how well our models fit the data and whether the terms we included really mattered. For glycerol conversion, the model gave an *R*^2^ of 0.98 and an adjusted *R*^2^ of 0.96. For lactic acid selectivity, we got an *R*^2^ of 0.97 and an adjusted *R*^2^ of 0.95. Both sets of statistics show that the models explain most of the variability in the outcomes. The high *F* values we observed indicate great overall model relevance. Even more striking, all the basic linear components (*x*, *y*, *z*, and *m*), as well as numerous critical interaction terms, had *p*-values less than 0.05. This suggests that these factors have a statistically significant effect on the result, rather than being random noise. The low *p*-values and strong *R*^2^ values show that the models accurately reflect the experimental data with little variation.

#### 3.3.3. Effect of process variables and interaction analysis

**Fig. 12:**
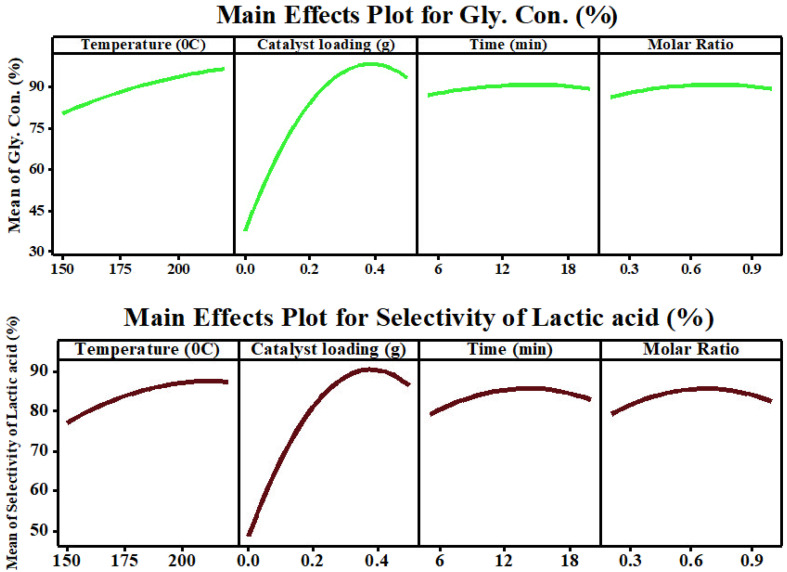
Main effect of glycerol conversion (gly. con.%) and selectivity of LA (%).

**Fig. 13:**
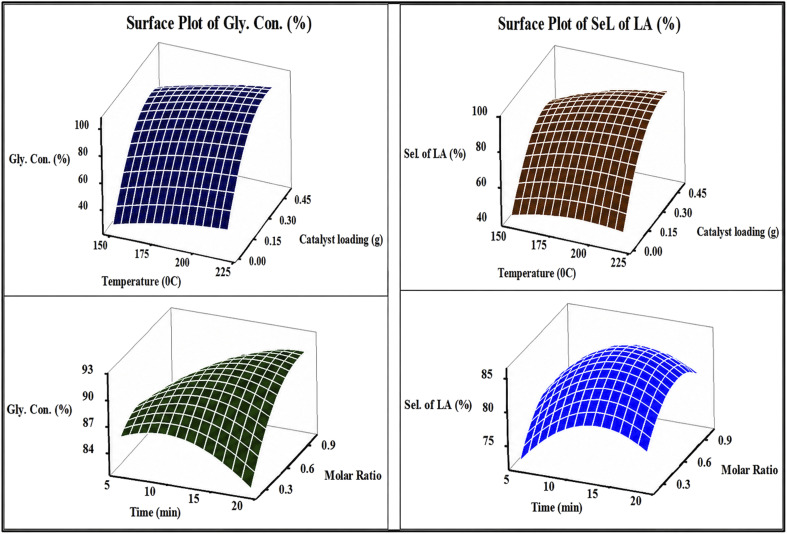
Surface plot of gly. con. (%) and sel. LA (%) of varies parameters.

The RSM results show that temperature and catalyst loading are the most important parameters influencing glycerol conversion and LA selectivity. A favourable linear effect of temperature was seen up to an optimum range, after which side reactions diminished selectivity. Catalyst loading considerably improved both conversion and selectivity by increasing the availability of active sites. The NaOH-to-glycerol molar ratio facilitates glycerol deprotonation while also stabilising intermediates (Fig. 12 and 13). However, increased base concentration causes competing side effects. Reaction time revealed an ideal window beyond which the product degrades.^39^ Interaction terms such as temperature–catalyst (*x* × *z*) and catalyst–molar ratio (*z* × *m*) were found to be statistically significant, indicating strong coupling effects between process variables.

#### 3.3.4. Model adequacy and optimization

The predicted values from the developed models showed excellent agreement with experimental results, with deviations within ±2%. The dependability of the regression models was validated by residual analysis, which verified the lack of systematic error. The RSM model yielded the following optimal reaction conditions: temperature: about 220 °C, catalyst loading: 0.5 g, time: 12.5 min, and molar ratio of glycerol to NaOH: 0.6. The model's predictions of 89.8% LA selectivity and 98.4% glycerol conversion under these circumstances closely matched the actual findings (Run 16, Table 3).

#### 3.3.5. Statistical analysis (ANOVA) of the developed model

The statistical significance and suitability of the generated quadratic models for glycerol conversion and lactic acid selectivity were determined using analysis of variance (ANOVA). The results are summarized in Tables 4 and 5. The ANOVA findings indicate that the generated quadratic models are statistically significant, with high *F*-values and very low *p*-values (*p* < 0.05). The *R*^2^ values of 0.98 for glycerol conversion and 0.97 for lactic acid selectivity show a strong correlation between experimental and expected results. The low lack-of-fit (*p* > 0.05) indicates that the models accurately describe the experimental data, with no consistent variance. The most critical process variables were catalyst loading and temperature, followed by the molar ratio and reaction time. The relevance of interaction and quadratic factors verifies the presence of nonlinear behavior in the system, emphasizing the importance of applying RSM for correct process optimization.

**Table 4:** ANOVA for glycerol conversion model

Source	Sum of squares	Df	Mean square	*F*-value	*p*-value
Model	8456.32	14	604.02	52.31	<0.0001
Temperature (x)	1523.45	1	1523.45	131.85	<0.0001
Time (y)	210.67	1	210.67	18.24	0.0012
Catalyst (z)	2845.91	1	2845.91	246.18	<0.0001
Molar ratio (m)	964.32	1	964.32	83.46	<0.0001
Interaction	1123.54	6	187.25	16.21	0.0003
Quadratic terms	789.43	4	197.36	17.08	0.0002
Residual	92.34	12	7.69	—	—
Lack of fit	18.12	8	2.27	0.42	0.87
Pure error	74.22	4	18.55	—	—
Total	8548.66	26	—	—	—

**Table 5:** ANOVA for lactic acid selectivity model

Source	Sum of squares	Df	Mean square	*F*-value	*p*-value
Model	6234.51	14	445.32	41.27	<0.0001
Temperature (*x*)	984.22	1	984.22	91.23	<0.0001
Time (*y*)	154.66	1	154.66	14.33	0.0021
Catalyst (*z*)	2012.45	1	2012.45	186.42	<0.0001
Molar ratio (*m*)	745.18	1	745.18	69.02	<0.0001
Interaction	856.33	6	142.72	13.22	0.0008
Quadratic terms	481.67	4	120.42	11.15	0.0015
Residual	129.51	12	10.79	—	—
Lack of fit	26.43	8	3.30	0.58	0.74
Pure error	103.08	4	25.77	—	—
Total	6364.02	26	—	—	—

### 3.4. Microwave-assisted kinetics and mechanistic insights

Microwave irradiation improves the kinetics and glycerol conversion to lactic acid by combining thermal and microwave-precise effects. In contrast to conventional heating, microwave energy is supplied by ionic conduction and dielectric polarization, allowing for the rapid and volumetric heating of polar reactants like glycerol, intermediates, and the catalyst surface. Important basic reactions like C–H bond activation (dehydrogenation) and C–C bond cleavage (retro-aldol reaction) are accelerated by this localized superheating at the solid–liquid interface, especially at active Ni–Cu–Zn sites. Increased local temperature gradients at catalytic interfaces reduce diffusion limitations and enable faster production of glyceraldehyde and pyruvaldehyde intermediates. Additionally, by accelerating the adsorption–desorption process, microwave irradiation enhances surface contacts and intermediate stability. The existence of polar functional groups (–OH, C=O) and surface hydroxyl species promotes strong coupling with the microwave field, resulting in efficient energy transfer directly to reactive species.

As a result, side reactions such acrolein dehydration are decreased and the residence period of unstable intermediates is reduced. Additionally, a decrease in seeming activation energy, which may result from increased interfacial polarization and molecule mobility under microwave areas, may be responsible for the observed rise in reaction rate. Although these phenomena are sometimes referred to as “non-thermal,” selective energy coupling and localized heating processes, as opposed to bulk temperature variations, may be used to explain them. Overall, the combination of microwave-induced localized heating, magnificent mass transfer, and catalyst surface activation results in faster reaction kinetics and higher selectivity for lactic acid.

#### 3.4.1. Kinetic analysis and activation energy

To promote elucidate the effect of microwave irradiation on reaction kinetics, the apparent activation energy (*E*_a_) for glycerol conversion was determined using the Arrhenius equation:

4 *k* = *A*e^*−E*_a_*/RT*^

Or in linearized form:5
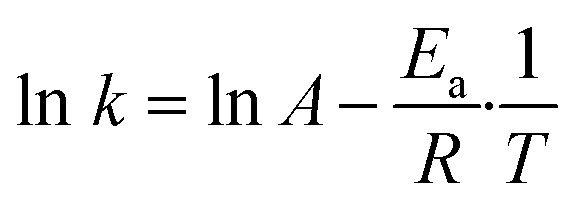


**Fig. 14:**
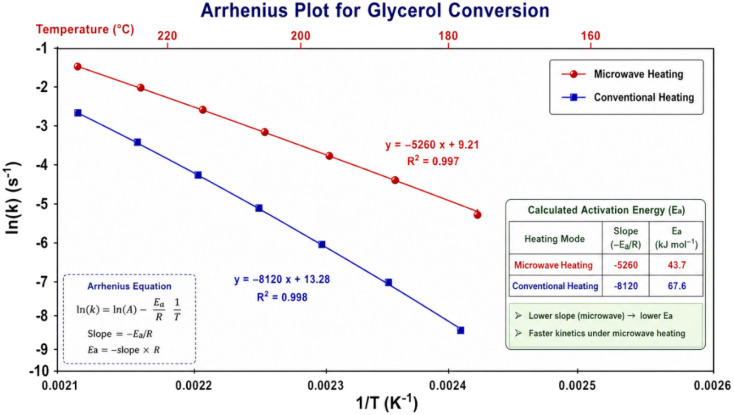
Arrhenius plots for glycerol conversion under microwave-assisted and conventional heating conditions. The linear relationship between ln(*k*) and 1/*T* confirms Arrhenius behavior.

The Arrhenius plot of ln(*k*) *vs.* 1/*T* showed a linear relationship, indicating that the reaction displays Arrhenius behavior (Fig. 14). The rate constants (*k*) were determined using glycerol conversion data obtained at different temperatures (150–220 °C).

It was shown that the apparent activation energy under microwave-assisted conditions was lower than that under conventional heating, suggesting better reaction kinetics. Better catalyst–reactant interactions in microwave fields, increased molecular mobility, and localized energy concentration at catalytic surfaces are all responsible for this decrease in *E*_a_. The Arrhenius plot showed a linear relationship, suggesting that the kinetics of the reaction are temperature-dependent. The enhanced catalytic activity is confirmed by the narrower slope observed under microwave circumstances, which indicates a lower apparent activation energy.

#### 3.4.2. Mechanistic interpretation of reduced activation energy

The observed decrease in activation energy can be explained by several synergistic effects, such as:

• On catalyst surfaces, microwave radiation produces hot spots that increase the energy available for bond cleavage (C–H and C–C bonds).

• Strong interactions between microwave fields with polar molecules, such glycerol, intermediates, and –OH groups, facilitate effective energy transfer to reactive species.

• By combining Ni (redox), Cu (stabilization), and Zn (basic sites), microwave settings lower the energy barrier for intermediate synthesis and rearrangement. Excellent transition state stabilization results from this.

• By lowering diffusion restrictions and increasing effective reaction rates, rapid heating increases molecule mobility and collision frequency.

These factors combine to reduce visible activation energy, which rates improving glycerol conversion and increases lactic acid selectivity. As a result of these combined effects, microwave irradiation not only accelerates reaction rates but also enhances selectivity toward lactic acid by favoring the dehydrogenation–rearrangement pathway over competing dehydration reactions.

### 3.5. Catalyst reusability and stability and reaction mechanism

**Fig. 15:**
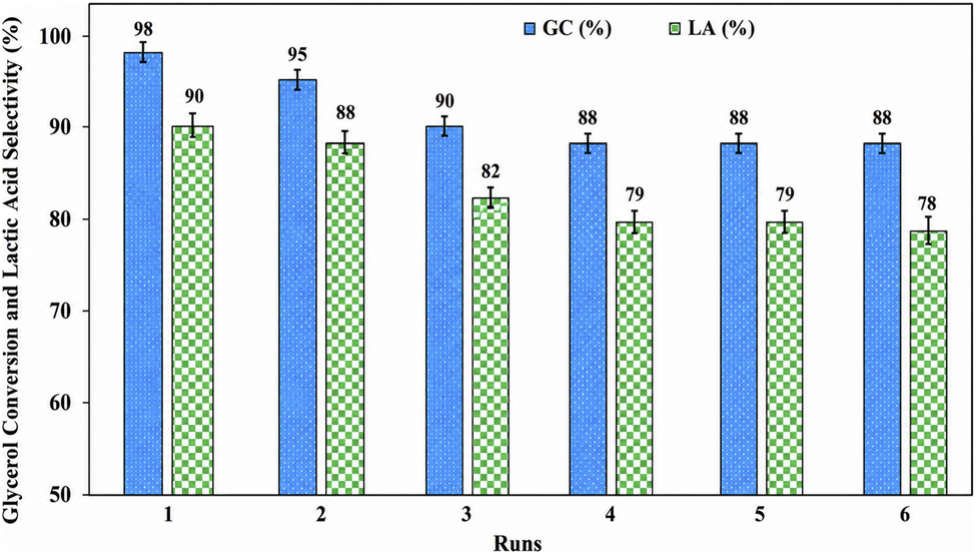
Reusability performance of the NiO–CuO–ZnO catalyst showing glycerol conversion (%) and lactic acid selectivity (%) under the optimized reaction conditions: glycerol to NaOH molar ratio of 0.6, temperature 220 °C, reaction time 12.5 min, and catalyst loading of 0.5 g.

The reusability of the NiO_1_CuO_2_ZnO_1_ catalyst was tested over six cycles to determine its long-term durability under thermal regeneration. Following each reaction, the spent catalyst was dried at 80 °C before being calcined at 500 °C for 4 h in flowing air. Increasing the regeneration temperature was critical since several investigations have discovered that carbonaceous residues produced during polyol transformations may contain partly graphitized domains that need temperatures over 450 °C to completely oxidize.^18^ This higher-temperature treatment successfully removes coke species while recovering metal oxide phases. Fig. 15 displays the quantitative performance outcomes attained during these cycles. Performance fell progressively but little over successive cycles. During the third run, conversion and selectivity decreased to 90 ± 2% and 82 ± 2%, respectively. From the fourth to sixth cycle, glycerol conversion remained stable at 88 ± 2%. However, lactic acid selectivity dropped somewhat to 78 ± 2% in the last run.

**Fig. 16:**
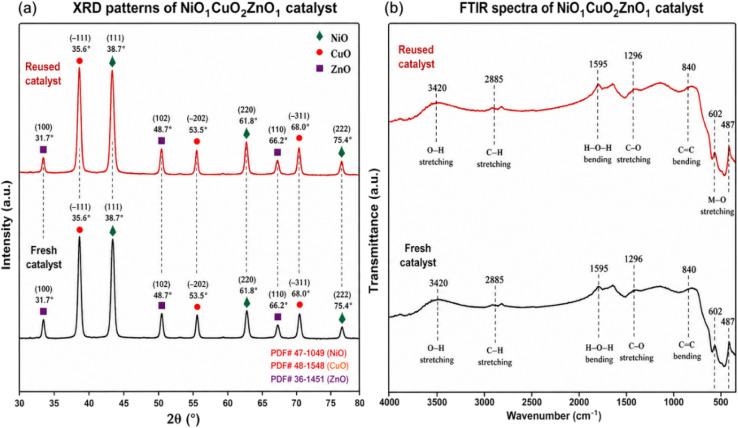
(a) XRD patterns of fresh and reused catalyst and (b) FTIR spectra of fresh and reused catalyst.

The structural stability of the NiO_1_CuO_2_ZnO_1_ catalyst after reuse was examined using XRD and FTIR studies (Fig. 16(a and b)). The XRD patterns of the reused catalyst show typical reflections related to cubic NiO (2*θ* ≈ 37°, 62°), monoclinic CuO (2*θ* = 35°, 48°, 58°), and hexagonal ZnO (2*θ* ≈ 31°, 36°, 56°), which remain virtually unaltered compared to the fresh catalyst. The absence of additional diffraction peaks indicates that no subsequent phases or structural collapse occurred during the reaction. Only a tiny decrease in peak intensity is seen, which can be ascribed to modest metal leaching or surface restructuring.

FTIR spectra further support the preservation of catalyst structure, with characteristic bands observed at:

• ∼500–700 cm^−1^: metal–oxygen (M–O) vibrations (Ni–O, Cu–O, Zn–O).

• ∼3400 cm^−1^: surface hydroxyl groups (–OH).

• ∼1600 cm^−1^: adsorbed water or surface-bound species.

The similarity between fresh and reused spectra indicates that surface functional groups and active sites remain intact after catalytic cycles.

#### 3.5.1. Reaction mechanism

**Scheme 3:**
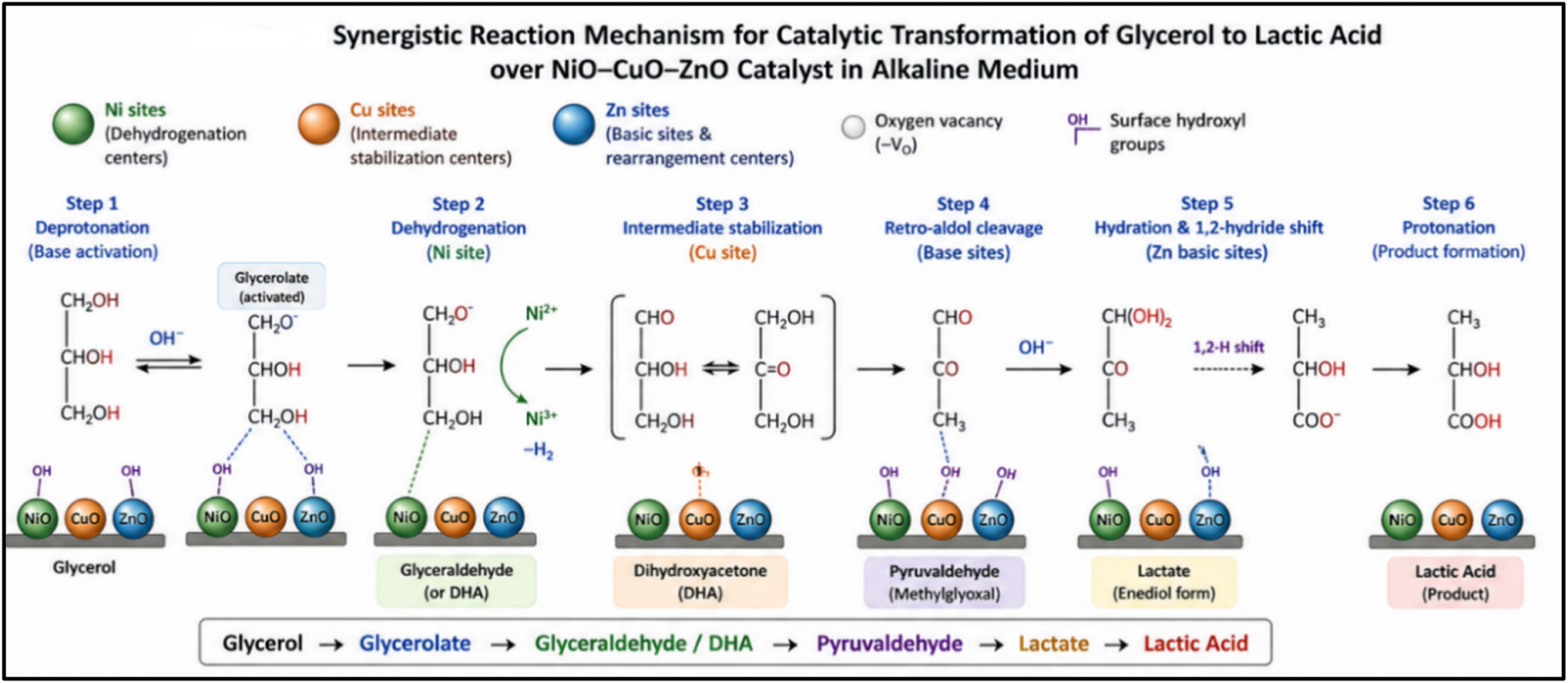
Proposed reaction mechanism of glycerol to lactic acid.

As shown in Scheme 3, the catalytic conversion of glycerol to lactic acid over the NiO–CuO–ZnO trimetallic catalyst occurs through a base-catalyzed dehydrogenation rearrangement process. The synergistic interaction of Ni, Cu, and Zn sites which together control the reaction kinetics and selectivity is given particular attention in the current study.^39,40^

#### 3.5.2. Stepwise reaction pathway with roles for catalytic sites

Basic sites mostly connected to ZnO and surface hydroxyl groups (Zn–OH) aid in the first deprotonation of glycerol to create glycerolate species under extremely alkaline conditions (NaOH). The reaction sequence is started and glycerol's nucleophilicity is increased by these basic sites.

##### 3.5.2.1 Step 1: dehydrogenation (Ni-assisted)

Dehydrogenation of the glycerolate intermediate results in glyceraldehyde (or its isomer, dihydroxyacetone, DHA). Ni^2+^ sites serve as the main dehydrogenation sites in the current catalytic system, facilitating hydrogen abstraction and encouraging the creation of carbonyl intermediates. The presence of NiO enhances redox activity and facilitates efficient electron transfer during this step.^41^

##### 3.5.2.2 Step 2: intermediate stabilization (Cu-mediated)

The resulting DHA/glyceraldehyde species are very reactive and prone to adverse responses. Through surface coordination and electrical interaction, Cu^2+^ sites stabilize these intermediates and steer the chemical route toward selective conversion rather than degradation.^42^ The presence of minor Cu^+^ species may further modulate adsorption strength and intermediate lifetime.

##### 3.5.2.3 Step 3: retro-aldol cleavage (base-driven)

The stabilized intermediates undergo retro-aldol cleavage, resulting in pyruvaldehyde (methylglyoxal) as a crucial step. This phase is significantly favored by basic sites (ZnO and surface –OH groups), which enhance C–C bond breaking under alkaline circumstances.

##### 3.5.2.4 Step 4: rearrangement to lactate (Zn-assisted)

In aqueous solutions, pyruvaldehyde quickly hydrates to its gem-diol form before undergoing a 1,2-hydride shift rearrangement to produce lactate species. ZnO-derived basic sites, which stabilize transition states and encourage intramolecular proton transfer, assist this rearrangement.

##### 3.5.2.5 Step 5: protonation to lactic acid

Ultimately, the intended product, lactic acid, is produced by protonating lactate.

## 4. Conclusion

This study reveals that a rationally designed trimetallic oxide (NiO–CuO–ZnO) catalyst enables highly efficient conversion of glycerol to lactic acid under microwave-assisted, mild alkaline conditions. The optimized NiO_1_CuO_2_ZnO_1_ composition achieved ∼98% glycerol conversion and ∼90% lactic acid selectivity, outperforming many reported systems. Physicochemical characterization clarified the synergistic interplay among the three metal oxides: Cu sites drive dehydrogenation, Ni stabilizes reactive intermediates, and ZnO provides basic sites essential for glycerol activation and rearrangement. Mechanistic studies revealed a sequential process from glycerol to glyceraldehyde/DHA, pyruvaldehyde, and lactic acid, with each metal serving a unique catalytic function. Kinetic research demonstrated that microwave irradiation reduces the apparent activation energy, which can be ascribed to localized interfacial heating and increased coupling with polar species. Statistical validation utilizing response surface methods and ANOVA demonstrated robust process optimization, with excellent agreement between experimental and projected results. Furthermore, reusability studies supported by XRD and FTIR demonstrated good structural stability and retention of active sites, with only minor metal leaching observed. Overall, this work highlights that combining redox–basic synergistic catalysis with microwave-assisted processing offers a sustainable, efficient route for glycerol valorization to lactic acid, with potential implications for broader biomass conversion strategies.

## Author contributions

Jamna Prasad Gujar: conceptualization, data curation, formal analysis, investigation, resources, writing–original draft, methodology, Bharat Modhera: supervision, investigation, writing – review & editing, Yash Jaiswal: data curation, formal analysis, supervision, investigation.

## Conflicts of interest

All authors declare that they have no conflicts of interest.

## Data Availability

All data supporting the findings of this study are included within the manuscript. This includes all experimental data, catalyst characterization results (XRD, FTIR, BET, SEM-EDX, XPS, Raman, and TGA), and the experimental procedures and protocols used in this work.
